# Numerical Study on the Variable-Temperature Drying and Rehydration of Shiitake

**DOI:** 10.3390/foods13213356

**Published:** 2024-10-23

**Authors:** Lizhe Zhang, Long Jiang, Meriem Adnouni, Sheng Li, Xuejun Zhang

**Affiliations:** Key Laboratory of Refrigeration and Cryogenic Technology of Zhejiang Province, Institute of Refrigeration and Cryogenics, Zhejiang University, Hangzhou 310027, China; zhanglizhe@zju.edu.cn (L.Z.);

**Keywords:** shiitake mushroom, variable-temperature convective drying, drying characteristics, rehydration

## Abstract

Variable-temperature convective drying (VTCD) is a promising technology for obtaining high-quality dried mushrooms, particularly when considering rehydration capacity. However, accurate numerical models for variable-temperature convective drying and rehydration of shiitake mushrooms are lacking. This study addresses this gap by employing a model with thermo–hydro and mechanical bidirectional coupling to investigate five dehydration characteristics (moisture ratio, drying rate, temperature, evaporation rate, and volume shrinkage ratio) and a drying load characteristic (enthalpy difference) during VTCD. Additionally, a mathematical model combining drying and rehydration is proposed to analyze the effect of VTCD processes on the rehydration performance of shiitake mushrooms. The results demonstrate that, compared to constant-temperature drying, VTCD-dried mushrooms exhibit moderate shrinkage rates and drying time (16.89 h), along with reduced temperature variation and evaporation rate gradient (Max. 1.50 mol/(m^3^·s)). VTCD also improves enthalpy stability, reducing the maximum drying load by 58.84% compared to 338.15 K constant-temperature drying. Furthermore, drying time at medium temperatures (318.15–328.15 K) greatly influences rehydration performance. This study quantitatively highlights the superiority of variable-temperature convective drying, offering valuable insights for optimizing the shiitake mushroom drying processes.

## 1. Introduction

Shiitake mushroom is a popular type of macrofungi with high nutritional value and health-promoting properties. It is the second most cultivated edible mushroom worldwide, accounting for about 25% of global mushroom production [1]. China produced over 11.88 million tons of shiitake mushrooms in 2020 [2]. However, this abundance presents a challenge, as fresh shiitake mushrooms have a high wet-basis moisture content of 85% to 90% [3]. Browning, lipid oxidation, and microbial deterioration occur when water interacts with the macromolecules of shiitake mushrooms [4]. The high moisture content necessitates significant drying efforts for preservation and further processing. In addition, dried shiitake mushrooms have a unique aroma and are more nutritious than fresh ones [5,6]. Thus, it is crucial to dry them in good time to improve their shelf life and quality.

Various drying methods have been studied for shiitake mushroom processing, including sun, convective, vacuum, microwave, infrared, heat pump, and freeze drying [7,8]. These approaches use different energy sources and equipment. Convective drying remains the most common drying approach due to its simplicity and practical advantages [4]. Heated air comes into direct contact with shiitake mushrooms, transferring heat and moisture by convection. Shiitake mushrooms are highly heat-sensitive. The most significant factor for shiitake mushroom convective drying is drying temperature, and its impacts on dehydration characteristics have been thoroughly researched. For instance, the shiitake mushrooms’ biological activity and heat-sensitive components are greatly influenced by drying temperature [9,10]. However, in most existing studies, the drying temperature for shiitake mushrooms remains constant, i.e., constant-temperature convective drying (CTCD). Recent research has discovered that shiitake mushrooms undergo complex physical and chemical changes during convective drying, which include aroma synthesis of enzymatic reactions and conversion between precursors and volatiles [11]. Therefore, the variable drying temperature is conducive to reducing the loss of heat-sensitive nutrients.

Variable-temperature convective drying (VTCD) optimizes the dried food quality by adjusting the drying temperature based on the food’s thermal characteristics [12]. It is an adaptive drying process that can achieve the multi-objective optimization of the quality of specific dried products [13]. Compared to some new drying technologies that require high costs, ordinary convective drying technology combined with a variable-temperature strategy is an efficient and cost-effective option [14]. Zhang et al. [15] found that VTCD not only effectively controlled browning in the freeze–thaw pretreatment of lotus root but also reduced the drying time and energy consumption of the process. Ayadi et al. [16] compared the spearmint drying curves of four CTCD processes with those of two VTCD processes, finding that the temperature-increasing VTCD was the best for drying time and operating cost. Recently, the performance of VTCD on shiitake mushrooms has also been investigated, and the results showed that VTCD was better than CTCD in terms of the shiitake mushroom rehydration ratio, heat pump COP, and drying time [17].

Optimizing VTCD processes is crucial for improving dried shiitake mushroom quality and system energy efficiency [17]. Existing studies mainly focus on drying parameters [16,18,19], neglecting the dynamic matching between energy supply and demand. The dynamic changes of heat load and moisture load in the drying process are particularly complicated because of the porosity and biological characteristics of shiitake mushrooms [4]. On the one hand, excessive energy supply not only accelerates nutrient loss [20], shrinkage [21], and hardening [22], but also results in energy waste. On the other hand, insufficient energy supply leads to insufficient dehydration and longer drying time [23]. Compared to CTCD, VTCD has more potential for achieving the dynamic matching between energy supply and demand by dividing a whole drying process into several sub-processes [24]. Energy demand is relatively steady in each sub-process. However, few studies have investigated the superiority of VTCD in supply–demand matching.

The optimization indices of VTCD processes are like those of CTCD processes. Dried mushrooms must be rehydrated before consumption. Rehydration represents the ability of dried mushrooms in solution to restore the properties of fresh ones [25]. The rehydration performance of dried food products is related to cell structural damage [26], composition change, and shrinkage during drying. Therefore, it is considered an essential quality attribute for dried shiitake mushrooms. García-Segovia et al. [27] performed shiitake mushroom CTCD at 50 °C. They compared the rehydration kinetics of shiitake mushrooms under different rehydration temperatures and pressures, discovering that the vacuum process had a faster rehydration velocity at lower temperatures than the atmospheric process. High rehydration temperature (80 °C) caused structural damage that prevented water absorption. Qiu et al. [28] investigated the water-holding capacity of rehydrated shiitake mushrooms in different CTCD conditions. Their study found significant changes in the rehydration ratio and water-holding capacity from 40 °C to 70 °C. This showed that drying temperature significantly affected the interior structure of rehydrated shiitake mushrooms. However, there is still a lack of research on the relationship between VTCD processes and shiitake mushroom rehydration kinetics.

In addition, current research focuses primarily on experimental investigations on shiitake mushroom VTCD [17,29]. It is time-consuming and expensive to study various VTCD processes due to the need for customized experiment systems. Moreover, the variations in material properties among mushrooms affect the analysis of the results. Numerical models can be utilized to investigate shiitake mushroom VTCD characteristics at a lower expense and in less time. However, accurate numerical models are still lacking for shiitake mushroom VTCD since it is a complex problem involving multiphysics processes, i.e., heat transfer, mass transfer, fluid flow, and shrinkage.

To fill the above gaps, this study aims to develop a comprehensive numerical model for shiitake mushroom VTCD by considering heat transfer, mass transfer, fluid flow, shrinkage, and interaction between physical field parameters. The model offers a cost-effective and time-saving alternative to traditional experimental methods. It investigates moisture ratio (MR), drying rate, temperature, evaporation rate, and volume shrinkage ratio of shiitake mushrooms during CTCD and VTCD. Additionally, the air enthalpy differences between the inlet and outlet of a chamber during CTCD and VTCD are compared to reveal energy demand patterns. Then, the relationship between VTCD processes and rehydration kinetics is examined, and a mathematical model combining drying and rehydration is established to analyze the effect of VTCD processes on the rehydration performance of shiitake mushrooms. Finally, the VTCD processes are evaluated based on the simulated water absorption capacity ratio to the total drying time to obtain an optimal drying process.

## 2. Materials and Methods

### 2.1. Materials and Instruments

**Figure 1:**
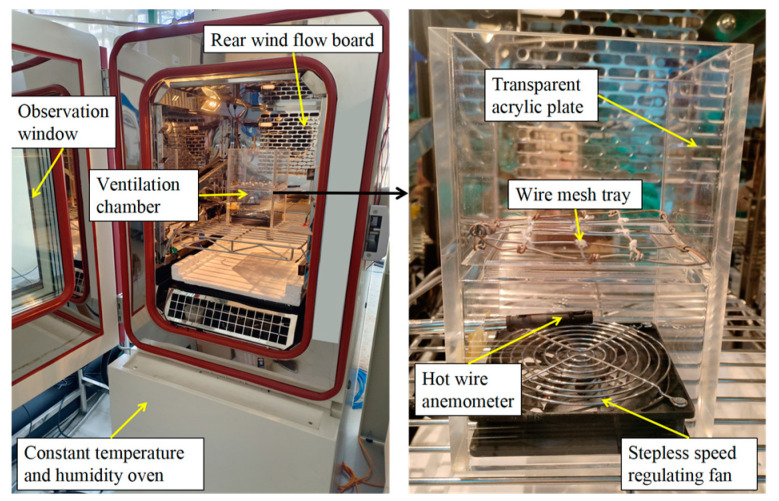
Experimental ventilation chamber.

Fresh shiitake mushrooms (*Lentinula edodes*) were harvested in September 2023 from Jinzifeng Edible Fungi Professional Cooperative in Qingyuan County, Zhejiang Province, China. The harvested mushrooms were immediately transported to the laboratory for refrigeration. Shiitake mushrooms’ diameter and height were 0.056 ± 0.004 m and 0.025 ± 0.002 m, respectively. The initial moisture content was 85.50 ± 2.66% (wet-basis). Samples of mushrooms were selected, with bright colors, fine and orderly gills, no abnormal external moisture, and no damage. Their stipes were trimmed, and their surfaces were cleaned up. A transparent acrylic ventilation chamber (Figure 1) was placed in a constant temperature and humidity oven (DHTH-100-0-P-SD). Its size was 0.12 m (length) × 0.12 m (height) × 0.2 m (width). The chamber bottom was equipped with a stepless speed regulating fan to control air velocity, and a small hole with a diameter of 0.015 m was on its side to place a hot wire anemometer. Hot air entered from the bottom and flowed out from the top. The shiitake mushrooms were in a wire mesh tray 0.1 m from the chamber bottom. The volume of shiitake mushrooms was measured using quartz sand (0.425–0.7 mm), a beaker, a funnel, and two graduated cylinders. During the drying and rehydration processes, the mass was measured using an electronic balance with an accuracy of 0.001 g. Distilled water, filter paper, and a thermostatic bath were used for rehydration. Furthermore, the initial dry basis mass of the mushrooms was determined using an electric drying oven.

### 2.2. Experimental Procedure

The shiitake mushroom drying experiments were conducted as follows: First, fresh mushrooms were removed from refrigeration and equilibrated to room temperature. Next, the ventilation chamber was preheated to a constant temperature and humidity to establish the drying environment. Then, the initial mass and volume of the mushrooms were measured using an electronic balance and quartz sand [30]. Mushrooms were placed cap-side up on the tray, and their mass and volume were recorded at 30 min intervals during the first three hours, followed by hourly measurements. After drying, the initial moisture content was determined using the 105 °C oven method [17].

The dried mushrooms were first weighed for the rehydration experiments, and distilled water (thirty times the mushroom mass) was prepared [31,32]. Subsequently, the water was heated to 60 °C in a thermostatic bath, after which the dried mushrooms were immersed for five hours. Mass was measured every 30 min after removing excess surface water with filter paper. Lastly, both the rehydration liquid and rehydrated mushrooms were dried to constant weight at 105 °C to determine their dry matter content. The dry matter holding capacity is the ratio between the dry matter weight in rehydrated mushrooms and the sum of dry matter in rehydration liquid and rehydrated mushrooms [33].

The Box–Behnken design (BBD) was used to fit the relationship between rehydration kinetic parameters and VTCD processes. The experimental factors and levels of the VTCD processes were set as shown in Table 1. There were 17 drying and rehydration experiments, formed by 12 factorial points and 5 center points. The drying temperature increased from 308.15 K to 338.15 K in stages. The relative humidity was 30%, and the drying was completed when the shiitake mushroom’s wet-basis moisture content was below 13%. In addition, the rehydration experiments of CTCD at 318.15 K and 338.15 K were supplemented.

A VTCD and two CTCD experiments were performed for drying model validation. For CTCD, the drying temperature (and relative humidity) were 333.15 K (20%) and 323.15 K (40%). For VTCD, the shiitake mushrooms were dried at 308.15 K, 318.15 K, and 328.15 K for 2 h, respectively, and then dried at 338.15 K until the drying process stopped. The wet-basis moisture content and volume shrinkage ratio of shiitake mushrooms during drying were calculated as Equations (1) and (2). Core temperatures were observed using needle-tip thermocouples inserted into the center of the shiitake mushrooms.

(1) $$X_{\mathrm{wb},t}=\frac{m_{t}-m_{0}\times \left(1-X_{\mathrm{wb},0}\right)}{m_{t}}$$

(2) $$SR_{\exp}=\frac{V_{0}-V_{t}}{V_{0}}$$

where *X*_wb,t_ and *X*_wb,0_ represent the shiitake mushroom wet-basis moisture content at a specific time during drying and before drying; *m*_t_ and *m*_0_ represent the shiitake mushroom mass at a specific time during drying and before drying, kg; *SR*_exp_ represents the experimental volume shrinkage ratio; *V*_t_ and *V*_0_ represent the shiitake mushroom volume at a specific time during drying and before drying, m^3^.

### 2.3. Variable-Temperature Convective Drying Numerical Model

**Figure 2:**
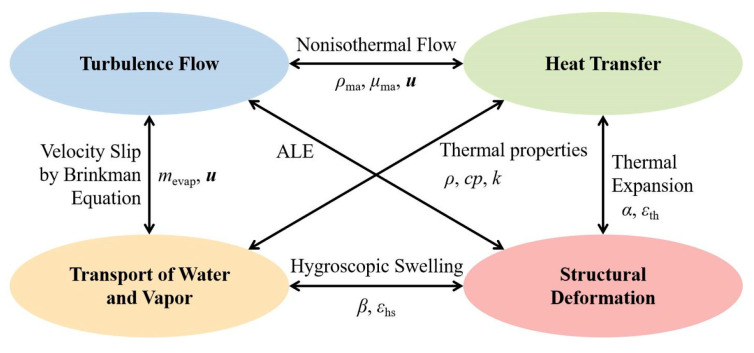
Multiphysics coupling in shiitake mushroom convective drying.

Shiitake mushroom convective drying is multiphysical [34], as shown in Figure 2. Hot air is supplied continuously and flows through the ventilation chamber as a turbulent flow. The liquid water inside the mushroom evaporates and diffuses to the surface, driven by temperature and concentration gradients. The liquid water then diffuses into the air as water vapor. Dehydration and thermal expansion lead to structural deformation of the solid matrix, promoting the movement of liquid and vaporized water. The mushroom’s altered shape modifies the air flow rate and turbulence region, affecting the heat and mass transfer. The arbitrary Lagrangian–Eulerian method (ALE) models the interaction between turbulence flow and structural deformation, addressing the coupling between fluid and structure. Temperature affects the physical properties of air and water.

Since the drying process is complicated, assumptions are made to develop the VTCD model:

1. Shiitake mushroom is an unsaturated wet porous medium with uniform initial temperature and moisture content, having viscoelastic mechanical properties.
2. Moisture only leaves the mushroom as water vapor. There is no liquid water on the mushroom’s surface.
3. Radiational, gravitational, and chemical energies are neglected.
4. The mushroom’s mass change is caused only by moisture loss.
5. The model is based on the Cartesian coordinate system, simplifying the 3D drying process to a 2D representation to more efficiently study the relative effects of different drying conditions.

#### 2.3.1. Governing Equations

The governing equations include moist air flow, energy, liquid water, and water vapor concentration, and stress–strain equations.

The Reynolds number is 19,505, according to the chamber size and air velocity. The algebraic turbulence model yPlus solves Reynolds-averaged Navier–Stokes (RANS) equations for momentum conservation (Equations (3) and (5)), a continuity equation for mass conservation (Equation (4)), and an algebraic equation for scaled wall distance (Equations (6)–(8)).

(3) $$\rho_{\mathrm{ma}}\frac{\partial u}{\partial t}+\rho_{\mathrm{ma}}\left(u\cdot \nabla \right)u=\nabla \cdot \left[-pI+K\right]$$

(4) $$\frac{\partial \rho_{\mathrm{ma}}}{\partial t}+\nabla \cdot \left(\rho_{\mathrm{ma}}u\right)=0$$

(5) $$K=\left(\mu_{\mathrm{ma}}+\mu_{T}\right)\left(\nabla u+{\left(\nabla u\right)}^{T}\right)-\frac{2}{3}\left(\mu_{\mathrm{ma}}+\mu_{T}\right)\left(\nabla \cdot u\right)I$$

(6) $$Re_{w}=\frac{\rho_{\mathrm{ma}}\left|u\right|l_{w}}{\mu_{\mathrm{ma}}}=\frac{\left|u\right|}{u_{\tau }}\cdot \frac{\rho_{\mathrm{ma}}u_{\tau }l_{w}}{\mu_{\mathrm{ma}}}=u^{+}l_{w}^{+}u^{+}=f\left(l_{w}^{+}\right)$$

(7) $$\nabla G\cdot \nabla G+\sigma_{w}G\left(\nabla \cdot \nabla G\right)=\left(1+2\sigma_{w}\right)G^{4}l_{w}=\frac{1}{G}-\frac{l_{\mathrm{ref}}}{2}$$

(8) $$\mu_{T}=\mu_{\mathrm{ma}}\left({\left(\frac{df}{dl_{w}^{+}}\right)}^{-1}-1\right)$$

where *ρ*_ma_ is the moist air density, kg/m^3^; ***u*** is the fluid velocity, m/s; *p* is the fluid pressure, Pa; ***K*** is the viscous stress tensor, Pa; *μ*_ma_ and *μ*_T_ are the moist air dynamic viscosity and turbulent viscosity, respectively, kg/(m·s); *u*_τ_ is the friction velocity, m/s; *l*_w_ is the wall distance, m; *G* is the reciprocal wall distance, 1/m; *σ*_w_ is the smoothing parameter.

The Brinkman equations are used in the porous media domain, as shown in Equations (9)–(11). The non-equilibrium approach explicitly speculates the evaporation rate, as shown in Equation (12) [35].

(9) $$\frac{\rho_{\mathrm{ma}}}{por_{\mathrm{sh}}\times S_{\mathrm{ma}}}\frac{\partial u}{\partial t}+\frac{\rho_{\mathrm{ma}}\left(u\cdot \nabla \right)}{por_{\mathrm{sh}}\times S_{\mathrm{ma}}}\frac{u}{por_{\mathrm{sh}}\times S_{\mathrm{ma}}}=\nabla \cdot \left[-pI+K\right]-\left(\frac{\mu_{\mathrm{ma}}}{\kappa_{\mathrm{ma}}}+\frac{C_{F}}{\sqrt{\kappa }}\rho_{\mathrm{ma}}\left|u\right|+\frac{Mn_{l}\cdot m_{\mathrm{evap}}}{po{r_{\mathrm{sh}}}^{2}\times {S_{\mathrm{ma}}}^{2}}\right)u$$

(10) $$\frac{\partial \left(por_{\mathrm{sh}}S_{\mathrm{ma}}\rho_{\mathrm{ma}}\right)}{\partial t}+\nabla \cdot \left(\rho_{\mathrm{ma}}u\right)=Mn_{l}\cdot m_{\mathrm{evap}}$$

(11) $$K=\frac{\mu_{\mathrm{ma}}}{por_{\mathrm{sh}}\cdot S_{\mathrm{ma}}}\left(\nabla u+{\left(\nabla u\right)}^{T}\right)-\frac{2}{3}\frac{\mu_{\mathrm{ma}}}{por_{\mathrm{sh}}\cdot S_{\mathrm{ma}}}\left(\nabla \cdot u\right)I$$

(12) $$m_{\mathrm{evap}}=K\left(aw\times c_{v,\mathrm{sat}}-c_{v}\right)por_{\mathrm{sh}}S_{\mathrm{ma}}$$

where *por*_sh_ is the mushroom porosity during shrinkage; *S*_ma_ is the gas phase saturation; *κ*_ma_ is the moist air permeability, m^2^ [36]; *C_F_* is the Forchheimer parameter, a dimensionless coefficient that accounts for the non-linear inertial effects in the flow through porous media at higher Reynolds numbers; *κ* is the absolute permeability of the porous medium; *Mn*_l_ is the liquid water molecular weight, kg/mol; *m*_evap_ is the evaporation rate, mol/(m^3^·s); *K* is the non-equilibrium evaporation rate constant, 1/s; *aw* is the mushroom water activity [37]; *c*_v,sat_ and *c*_v_ are the saturated vapor concentration and vapor concentration, mol/m^3^.

The work performed by pressure changes and viscous dissipation in the fluid is ignored to simplify the energy equations. Equation (13) defines the temperature field of free airflow. Water vapor concentration affects the thermal characteristics of moist air, i.e., density, specific heat capacity, and thermal conductivity. The thermal convection velocity field utilizes the solution of the flow equation. The thermal properties in the cumulative and conduction terms of the porous media domain’s energy equation are weighted by phase volume fractions. The thermal properties of the first convection term apply to liquid water and moist air in porous media. Moreover, the latent heat absorbed by liquid water evaporation is added as the source term, as defined by Equation (14).

(13) $$\rho_{\mathrm{ma}}cp_{\mathrm{ma}}\frac{\partial T}{\partial t}+\rho_{\mathrm{ma}}cp_{\mathrm{ma}}u\cdot \nabla T-\nabla \cdot \left(k_{\mathrm{ma}}\nabla T\right)=0$$

(14) $$\rho_{\mathrm{eff}}cp_{\mathrm{eff}}\frac{\partial T}{\partial t}+\rho_{f}cp_{f}u_{f}\cdot \nabla T-\nabla \cdot \left(k_{\mathrm{eff}}\nabla T\right)=-H\times Mn_{l}\times m_{\mathrm{evap}}$$

where *cp*_ma_ is the moist air heat capacity, J/(kg·K); *T* is the temperature, K; *k*_ma_ is the moist air thermal conductivity, W/(m·K); *ρ*_eff_, *cp*_eff_, and *k*_eff_ are the effective volumetric density (kg/m^3^), heat capacity (J/(kg·K)) and thermal conductivity (W/(m·K)) for all phases [37]; *ρ*_f_, *cp*_f_ and ***u***_f_ are the mobile fluid combined density (kg/m^3^), heat capacity (J/(kg·K)), and velocity (m/s); *H* is the latent heat, J/kg.

Capillary suction is the basis for liquid water diffusion, and Darcy’s law is used to determine convective transfer velocity. The promoting effect of solid shrinkage on liquid water mass transfer is considered. Liquid water decreases due to evaporation. Thus, Equation (15) represents the liquid water mass equation.

(15) $$\frac{\partial c_{l}}{\partial t}+\nabla \cdot \left(-D_{\mathrm{cap}}\nabla c_{l}\right)+\left(-\frac{\nabla p\times \kappa_{l}}{S_{l}\times por_{\mathrm{sh}}\times \mu_{l}}\right)\cdot \nabla c_{l}+u_{s}\nabla c_{l}=-m_{\mathrm{evap}}$$

where *c*_l_ is the liquid water concentration, mol/m^3^; *D*_cap_ is the capillary diffusivity, m^2^/s [38]; *κ*_l_ is the liquid water permeability, m^2^ [36]; *S*_l_ is the liquid water saturation; *μ*_l_ is the liquid water viscosity, kg/(m·s).

Equations (16) and (17) represent water vapor transportation in porous media and air-free flow, respectively. Equation (18) illustrates that the water vapor diffusion in porous media is related to its diffusion coefficient in free air and the porous media porosity [39,40]. The convection term includes the water vapor migration caused by the moving velocity of the solid matrix [37]. Equation (19) expresses the diffusion coefficient’s main determinants.

(16) $$\frac{\partial c_{v}}{\partial t}+\nabla \cdot \left(-D_{\mathrm{eff}}\frac{Mn_{a}}{Mn_{\mathrm{ma}}}\nabla c_{v}\right)+\left(\frac{u}{S_{\mathrm{ma}}por_{\mathrm{sh}}}-\frac{Mn_{a}D_{\mathrm{eff}}}{Mn_{\mathrm{ma}}\rho_{\mathrm{ma}}}\nabla \rho_{\mathrm{ma}}\right)\cdot \nabla c_{v}+u_{s}\nabla c_{v}=m_{\mathrm{evap}}$$

(17) $$\frac{\partial c_{v}}{\partial t}+\nabla \cdot \left(-D_{\mathrm{va}}\nabla c_{v}\right)+u\cdot \nabla c_{v}=0$$

(18) $$D_{\mathrm{eff}}=D_{\mathrm{va}}\times po{r_{\mathrm{sh}}}^{\frac{4}{3}}{S_{\mathrm{ma}}}^{\frac{10}{3}}$$

(19) $$D_{\mathrm{va}}=2.2\times 10^{-5}\times \frac{p_{0}}{p}{\left(\frac{T}{T_{0}}\right)}^{\frac{3}{2}}$$

where *D*_eff_ is the effective vapor diffusivity, m^2^/s; *Mn*_a_ is the dry air molecular weight, kg/mol; *D*_va_ is the air-vapor diffusivity, m^2^/s.

The shiitake mushroom displacements, mechanical stresses, and strains are computed using Equations (20)–(25).

(20) $$\rho_{\mathrm{eff}}\frac{\partial^{2}U}{\partial t^{2}}=\nabla \cdot {\left(F\sigma \right)}^{T},\;F=I+\nabla U$$

(21) $$\sigma =\sigma_{\mathrm{inel}}+J_{i}F_{\mathrm{inel}}^{-1}\left(C:\varepsilon_{\mathrm{el}}\right)F_{\mathrm{inel}}^{-T},\;\varepsilon_{\mathrm{el}}=\frac{1}{2}\left(F_{\mathrm{el}}^{T}F_{\mathrm{el}}-I\right),F_{\mathrm{el}}=FF_{\mathrm{inel}}^{-1}$$

(22) $$\sigma_{\mathrm{inel}}=\sigma_{0}+\sigma_{\mathrm{ext}}+\sigma_{q}$$

(23) $$\varepsilon =\frac{1}{2}\left[{\left(\nabla U\right)}^{T}+\nabla U+{\left(\nabla U\right)}^{T}\nabla U\right]$$

(24) C=C(E,ν)

(25) $$\rho_{\mathrm{eff}}=\rho_{s}\left(1-por_{\mathrm{sh}}\right)+\rho_{l}por_{\mathrm{sh}}S_{l}+\rho_{\mathrm{ma}}por_{\mathrm{sh}}S_{\mathrm{ma}}$$

where ***U*** is the structural displacement, m; *F* is the deformation gradient tensor; *σ* is the second Piola–Kirchhoff stress, Pa; *ε*_el_ is the elastic strain; *σ*_0_, *σ*_ext_, and *σ*_q_ are the initial stress, external stress, and viscoelastic stress, respectively, Pa; ***C*** is the constitutive tensor; *E* is the elastic modulus, Pa; *ν* is the Poisson’s ratio.

Ignoring the effects of shrinkage on heat and mass transfer reduces simulation accuracy [41]. However, the linear elastic model is only valid for small deformations [42]. The generalized Maxwell model for viscoelastic material is the most common method for predicting the stress–strain relationship during drying of different foods, such as strawberries [43], carrots [44], and pumpkins [45]. The stress relaxation curves of shiitake mushrooms [46] are used to model the relationship between generalized Maxwell parameters and drying conditions, which is provided in the Supplementary Materials.

The total strain also includes thermal and hygroscopic strains, defined by Equations (26) and (27), respectively. Equation (28) determines the hygroscopic expansion coefficient by fitting experimental data.

(26) $$\varepsilon_{\mathrm{th}}=\alpha \left(T\right)\left(T-T_{\mathrm{ref}}\right)$$

(27) $$\varepsilon_{\mathrm{hs}}=\beta Mn_{l}\left(c_{l}-\frac{S_{l,0}\times por_{\mathrm{sh}}\times \rho_{l}}{Mn_{l}}\right)$$

(28) $$\beta =9\times 10^{-4}\ln\left(15+X_{\mathrm{db}}\right)$$

where *ε*_th_ and *ε*_hs_ are the thermal strain and hygroscopic strain; *α* is the thermal expansion coefficient, 1/K; *T*_ref_ is the reference temperature, K; *β* is the hygroscopic expansion coefficient, m^3^/kg; *X*_db_ is the dry basis moisture content.

#### 2.3.2. Initial and Boundary Conditions

The model parameters are listed in Table 2. The non-equilibrium evaporation rate constant *K* depends on the ambient fluid, the convective heat transfer coefficient, etc. While independent methods of measuring *K* for a hygroscopic material are still lacking, pure water evaporation was estimated to be of the order of 1 1/s [47,48]. The drying process lowers moisture content while increasing glass transition temperature. However, shiitake mushrooms with 15% wet-basis moisture content have a glass transition temperature below −40 °C [49], much lower than the drying temperature. Therefore, shiitake mushrooms are rubbery and show viscoelastic behaviors during drying. The Poisson’s ratio is taken as 0.49 according to [22]. The initial water saturation is calculated from the initial moisture content, solid matrix density, and liquid water density [50]. The initial conditions are listed in Table 3.

**Figure 3:**
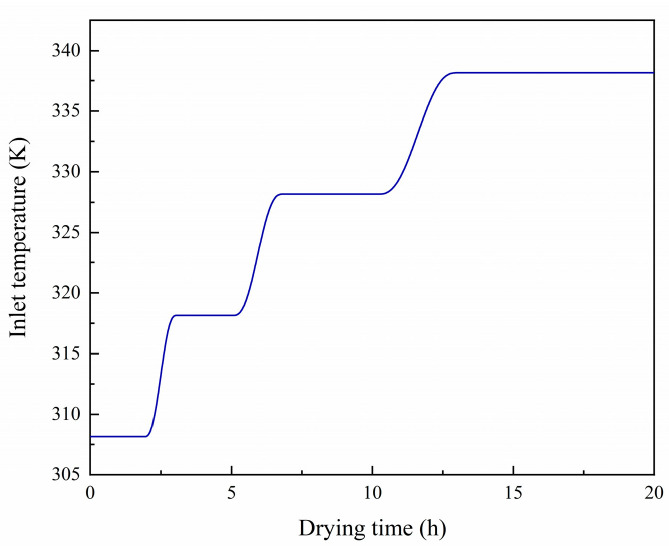
VTCD for drying characteristics analysis.

The Dirichlet boundary condition is adopted at the inlet of the ventilation chamber. The inlet flow is fully developed, where the average air state parameters (temperature, relative humidity, and air velocity) are consistent with the drying conditions. A piecewise function, as shown in Figure 3, defines the drying temperature of VTCD for drying characteristics analysis. The shiitake mushroom is dried at 308.15 K, 318.15 K, and 328.15 K for 2.4 h, 3.3 h, and 5.6 h, respectively, and then dried at 338.15 K until the moisture content reaches 13% [17]. The temperature curve is smoothed using the continuous second derivative. The ventilation chamber walls are no-slip, adiabatic, and have no mass flux. The outlet relative pressure is 0 Pa. There are no liquid water flux and load constraints on mushroom’s boundaries.

#### 2.3.3. Numerical Solution

The model is solved using COMSOL Multiphysics software. The turbulent flow algebraic yPlus, heat transfer in fluids, transport of diluted species, and solid mechanics interfaces are used to compute the velocity and pressure, temperature, concentration of water vapor and liquid water, and strain inside the shiitake mushroom, respectively. Furthermore, the non-isothermal flow multiphysics coupling is used to simulate fluid flows whose properties are temperature-dependent. Moreover, thermal expansion and hygroscopic swelling multiphysics coupling are added to simulate the internal strain caused by temperature and moisture changes. The solver stops when the average wet base moisture content is less than 13%. In each time step, the solutions are assumed to be converged when the relative toleration is less than 0.01.

Mesh independence study is an essential step in numerical simulation. Three grids are tested for key variables, relative errors, and grid convergence indices (GCI) [52]. The wet-basis moisture content after 10 h VTCD drying is used as the key variable. As shown in Table 4, both GCI_12_ and GCI_23_ are less than 5%. Grid2 is used to solve the model in order to save computing time.

**Figure 4:**
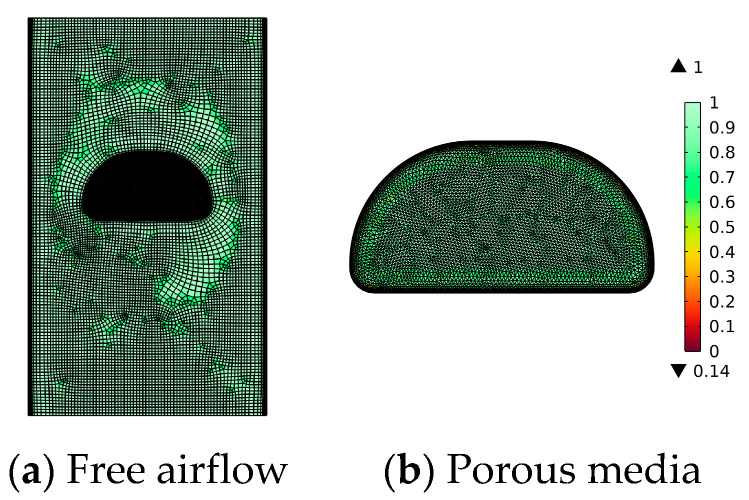
Numerical meshing. (The color legend refers to the skewness in the grid cell quality).

As shown in Figure 4, the meshing is built using the COMSOL predefined “free quadrilateral” with an “extra fine” mesh element size for the air-free flow region and “free triangular” with a “extremely fine” mesh element size for the porous medium domain, calibrated for fluid dynamics. Eight boundary layers are added to the border of the mushroom and chamber walls (adjustment factor 1.2). A moving mesh governs the space frame, and the air-free flow domain is designated as the deformation domain. In the transient study, select automatic remeshing to prevent mesh cell inversion and maintain mesh quality, ensuring calculation stability and accuracy.

### 2.4. Shiitake Mushroom Rehydration Model after Variable-Temperature Convective Drying

#### 2.4.1. Rehydration Kinetics Modelling

Peleg [53] proposed a two-parameter, nonexponential empirical model to fit sorption curves for rice soaked in water. This model has been widely used to describe the rehydration behavior of food during different drying processes [54,55,56], and it fits well with the experimental data of rehydration kinetics of dried edible fungi, such as boletus edulis mushroom [57], morel [58], and shiitake mushroom [27]. Therefore, Peleg model is used to describe the rehydration kinetics of shiitake mushrooms after VTCD:

(29) $$X_{\mathrm{db},t}=X_{\mathrm{db},d}+\frac{t}{K_{1}+K_{2}\times t}$$

where *X*_db,t_ and *X*_db,d_ are the dry basis moisture content during and before rehydration, respectively; *t* is the rehydration time, min; *K*_1_ is the Peleg rate constant, min; *K*_2_ is the Peleg capacity constant.

#### 2.4.2. Rehydration Indices

Rehydration comprises three simultaneous processes: the imbibition of water into dried material, the swelling of the rehydrated products, and the leaking of soluble solids [59]. Because the rehydration ratio measures the relative mass increase in dried material rehydrated in water, it provides no information regarding the matrix’s ability to recover water lost during drying. Therefore, the rehydration ratio cannot be used to explain the material’s rehydration characteristics after drying individually. Lewicki [60] proposed three indices to calculate the rehydration ability of dried foods. The water absorption capacity (WAC), dry matter holding capacity (DHC), and rehydration ability (RA) are calculated as follows:

(30) $$WAC=\frac{m_{r}\left(1-s_{r}\right)-m_{d}\left(1-s_{d}\right)}{m_{0}\left(1-s_{0}\right)-m_{d}\left(1-s_{d}\right)}$$

(31) $$DHC=\frac{m_{r}s_{r}}{m_{d}s_{d}}$$

(32) RA=WAC⋅DHC

where *m* and *s* represent a sample’s mass and percent dry matter content (0, d, r–initial, dried, rehydrated).

#### 2.4.3. Relationship with Drying Numerical Model

First, the connection between VTCD processes and shiitake mushroom’s rehydration kinetics is established. The rehydration calculation module is then added to the shiitake mushroom VTCD numerical model, creating a mathematical model combining drying and rehydration. This integration enables the examination of the gradient change in drying characteristics (difficult to measure in experiments) and its relationship to rehydration effectiveness, as well as the demonstration of VTCD technology’s advantages. The derivation of the formula is shown in Appendix A.

### 2.5. Statistical Analysis

Origin 2018 and Design Expert 11 were used for statistical analysis. Drying and rehydration experiments were conducted in triplicate. The drying model’s accuracy was evaluated using statistical indicators, including the coefficient of determination (R^2^), root mean square error (RMSE), and mean absolute error (MAE). Non-linear regression analysis of the experimental data was conducted to obtain the kinetic parameters *K*_1_ and *K*_2_ of shiitake mushroom rehydration processes after different VTCD processes. Statistical parameters include standard error (SE), reduced chi-square (χ^2^), and adjusted R-square (Adj. R^2^). Analysis of variance (ANOVA) for the model of the Peleg rate constants was employed to identify significant differences (*p* < 0.05). Further, a polynomial relationship between rehydration kinetic parameters and drying time at each temperature in VTCD processes is obtained. The calculation and meanings of the statistical parameters are provided in the Supplementary Materials.

## 3. Results and Discussion

### 3.1. Dehydration Characteristics

#### 3.1.1. Drying Model Validation

**Figure 5:**
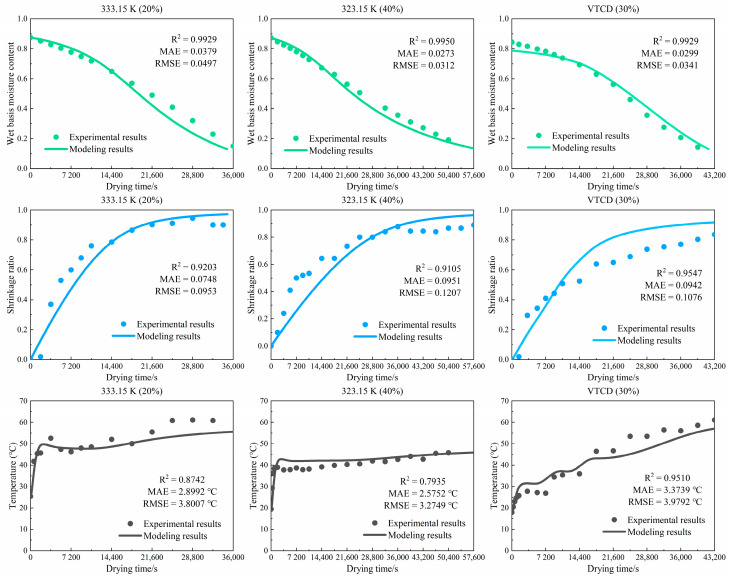
Experimental and numerical results of the wet-basis moisture content, volume shrinkage ratio, and core temperature under different shiitake mushroom drying processes.

Figure 5 shows the numerical and experimental results of the wet-basis moisture content, volume shrinkage ratio, and core temperature of the shiitake mushroom. The numerical results of the drying model are very close to the experimental results. The average coefficients of determination (R^2^) are 0.9936, 0.9285, and 0.8729 for the wet-basis moisture content, volume shrinkage ratio, and core temperature, respectively. The mean absolute errors (MAEs) are 0.0226, 0.0880, and 2.9494 K for the wet-basis moisture content, volume shrinkage ratio, and core temperature, respectively. Moreover, the root mean square errors (RMSEs) are 0.0383, 0.1079, and 3.6849 K for the wet-basis moisture content, volume shrinkage ratio, and core temperature, respectively. Accordingly, this model simulates the shiitake mushroom convective drying process accurately. This model investigates the shiitake mushroom dehydration and drying load characteristics during VTCD.

#### 3.1.2. Moisture Ratio and Drying Rate of Shiitake Mushrooms

**Figure 6:**
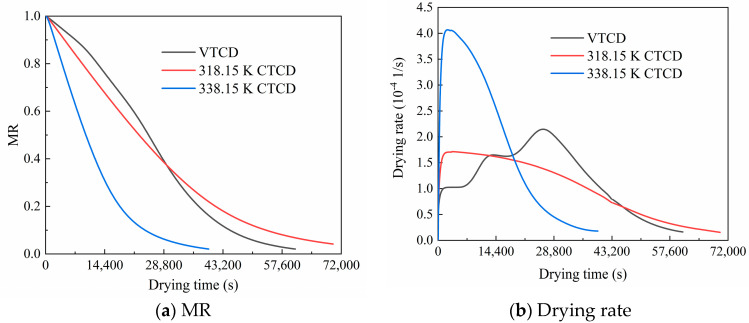
MR and drying rate of shiitake mushrooms in VTCD and CTCD processes.

In industrial applications, the temperature for CTCD of shiitake mushrooms typically ranges from 318.15 K to 338.15 K for efficient moisture removal while preserving quality. Therefore, the numerical model simulates a VTCD process and two CTCD processes at 318.15 K and 338.15 K. In the VTCD process, drying occurs at 308.15 K, 318.15 K, and 328.15 K for 2.4 h, 3.3 h, and 5.6 h, respectively, before continuing at 338.15 K until completion [17]. The drying relative humidity is 30%. MR is an important dimensionless parameter to quantify variation in the moisture content of shiitake mushrooms during drying. The drying rate refers to the derivative of dry basis moisture content with respect to time. As illustrated in Figure 6, although the MRs of the three drying processes all decrease from 1 to about 0.03, the MR of the VTCD process shows the lowest decreasing rate at the early drying stage and is the largest in the first eight hours.

Furthermore, the drying rates of the two CTCD processes increase sharply at the early drying stage and decrease continuously after reaching the peak. A higher drying air temperature results in a faster drying rate and a shorter drying time. This is due to increased ambient heat supply to the product and accelerated water mobility within the mushroom. These findings are consistent with the literature on drying [16]. Meanwhile, the drying rate of the VTCD process is relatively steady. Although the final drying temperature of VTCD reaches 338.15 K, the maximum drying rate (0.0002 1/s) is about half that of 338.15 K CTCD (0.00041 1/s), which is close to the 318.15 K CTCD (0.00017 1/s).

#### 3.1.3. Temperature of Shiitake Mushrooms

**Figure 7:**
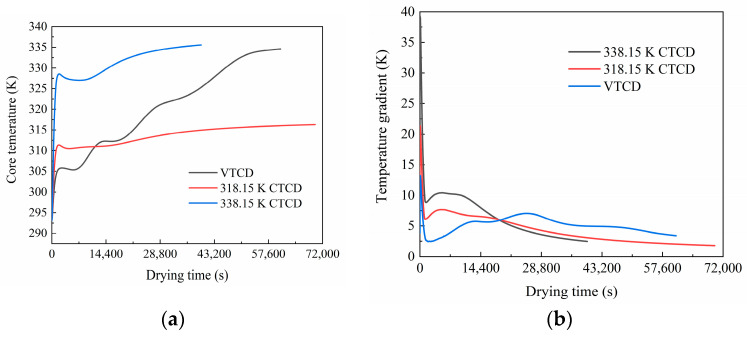
Core temperature (**a**) and temperature gradient (**b**) of shiitake mushrooms in VTCD and CTCD processes.

Figure 7 illustrates the core temperature and temperature gradient. The bottom of the shiitake mushroom reaches the drying temperature the fastest, so the initial temperature gradient during drying is approximately the difference between the drying temperature and the ambient temperature. There are apparent temperature gradients inside the shiitake mushroom in the CTCD processes. As heat is transferred to the interior of the shiitake mushroom, the temperature gradient of the mushroom decreases rapidly, but it still maintains relatively high during the initial drying stage (the temperature gradients of 338.15 K CTCD, 318.15 K CTCD, and VTCD can reach 10.41 K, 7.68 K, and 3.12 K, respectively). For CTCD, the core temperatures rise rapidly in the first hour, approach their drying temperatures, and then drop slightly. The reason is that the evaporation rate in the internal region is significant, and the heat consumed by evaporation exceeds that transferred from the surface. The evaporation rate slows down in the middle of drying, and the temperature inside the mushroom rises again. Two hours after the start of drying, the temperature gradient of CTCD gradually decreases. However, the temperature rises uniformly in the VTCD process, and the shiitake mushroom temperature gradient is relatively small and stable. Since the endpoint of the drying experiment is a wet-basis moisture content of 13% rather than 0%, moisture evaporation and heat absorption continue throughout the process. Consequently, the core temperatures of the shiitake mushrooms remain below the drying temperatures. The shiitake mushroom temperature distribution in the VTCD and CTCD processes after drying for 1, 4, and 8 h and at the last moment are displayed in Appendix B.

#### 3.1.4. Evaporation Rate of Shiitake Mushrooms

**Figure 8:**
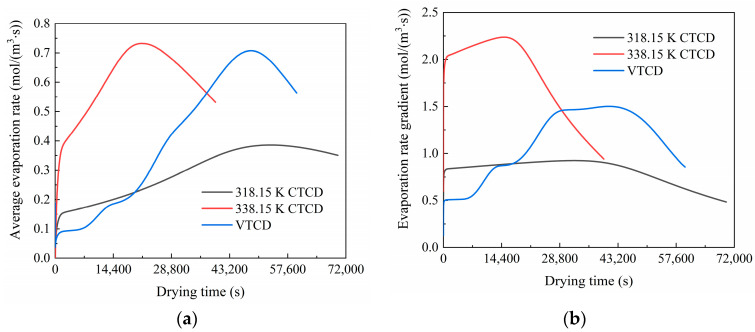
Average evaporation rate (**a**) and evaporation rate gradient (**b**) of shiitake mushrooms in VTCD and CTCD processes.

The average evaporation rates (molar amount of evaporation water per unit volume and unit time) and evaporation gradients are shown in Figure 8. The evaporation rate changes considerably in the CTCD processes. In the early drying stage, the evaporation rate of VTCD is the lowest. The peak average evaporation rates of 338.15 K CTCD, 318.15 K CTCD, and VTCD reach 0.73, 0.39, and 0.71 mol/(m^3^·s), respectively, while the peak evaporation rate gradients reach 2.24, 0.92, and 1.50 mol/(m^3^·s), respectively. Therefore, VTCD can simultaneously achieve a high and low evaporation rate gradient in the later drying stages. During drying at increasing temperatures, the stimulations during temperature changes generate an acceleration of the phenomena of evaporation, which is also found in spearmint [16]. The distribution of shiitake mushroom evaporation rate in the VTCD and CTCD processes is shown in Appendix B. The results show that the variation in the evaporation rate in the VTCD process is the smallest at the early stage. Water evaporation mainly occurs in the external region of the shiitake mushroom, exhibiting a significantly higher evaporation rate than the internal region. The evaporation rate in the external region gradually decreases with the decrease in moisture content.

#### 3.1.5. Volume Shrinkage Ratio of Shiitake Mushrooms

**Figure 9:**
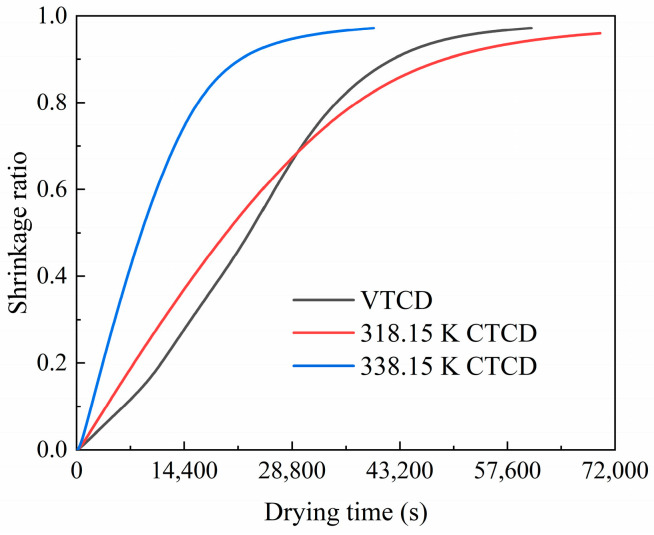
Shrinkage ratio of shiitake mushrooms in VTCD and CTCD processes.

The shrinkage ratio of shiitake mushrooms in the VTCD and CTCD processes are compared by calculating their volumetric strain, as displayed in Figure 9. The shrinkage ratios increase gradually and are more than 0.9 at the final dried state. Although the shrinkage ratio of the VTCD process is moderate after drying, it maintains the smallest for the first eight hours. It is noted that increased shrinkage ratios can reduce rehydration capacity because of surface cracking, a phenomenon that becomes apparent during the drying process [61]. Therefore, VTCD can mitigate this impact.

#### 3.1.6. Enthalpy Difference between the Inlet and Outlet of the Drying Chamber

**Figure 10:**
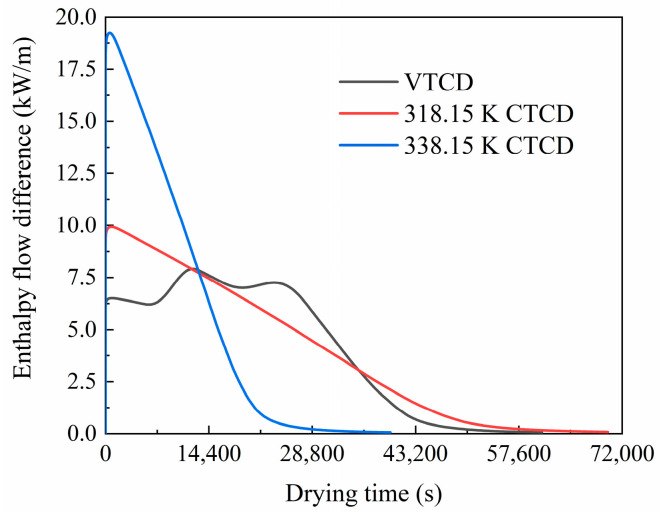
Air enthalpy difference between the inlet and outlet of the drying chamber in VTCD and CTCD processes.

The humid air enthalpy is integrated into the inlet and outlet boundary to compare heat and moisture loads of the drying chamber in VTCD and CTCD processes. The drying load in kW/m represents the energy transfer rate per unit width in the 2D plane, which is consistent with the Cartesian coordinate system. This unit is used because the simulation represents a vertical 2D slice of the drying chamber. The energy transfer rate for the entire system can be obtained by integrating over the width or applying a suitable geometric correction if necessary. This approach is used for relative comparisons under different drying conditions. The enthalpy flow curves changing with drying time are shown in Figure 10. The total drying time of the VTCD process is between those of the two CTCD processes. The drying load of the VTCD process shows better stability compared to those of CTCD processes, especially at the early drying stage. The maximum drying loads are 19.24 kW/m (338.15 K CTCD), 9.93 kW/m (318.15 K CTCD), and 7.92 kW/m (VTCD), respectively. The maximum drying load of VTCD is 41.16% of that of 338.15 K CTCD. The VTCD process exhibits the narrowest range of heat and moisture load fluctuations, which is advantageous for the steady and energy-efficient operation of energy supply equipment like heat pumps.

### 3.2. Rehydration Processes

#### 3.2.1. Rehydration Kinetics after Variable-Temperature Convective Drying

**Figure 11:**
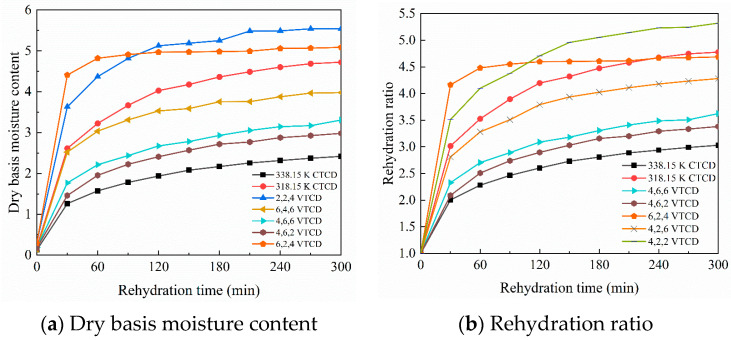
Experimental results of rehydration kinetics of shiitake mushrooms under different drying processes.

Figure 11a,b show the experimental rehydration kinetics of shiitake mushrooms after different drying processes. The numbers before ‘VTCD’ in the legend represent the drying time at 308.15 K, 318.15 K, and 328.15 K, respectively. Two CTCD processes and thirteen VTCD processes are performed, as described in Section 2.2. For the simplicity of the graphical display, only five representative VTCD data points are retained, and the other eight sets of results are close to that of 318.15 K CTCD. Within the first 30 min of rehydration, all mushrooms absorb water rapidly, which raises the dry basis moisture contents. Over the next several hours, the water absorption rates gradually decrease until the equilibrium moisture contents are reached. Surface and capillary suction are the main causes of the first stage’s rapid moisture absorption.

Upon wetting, the surface moisture concentration rises to saturation immediately, and moisture movement is restricted within the material [62]. The driving force for water transfer diminishes as the rehydration process approaches equilibrium [63]. The initial moisture contents of shiitake mushrooms are unknown during drying experiments because they are determined by drying the mushrooms and rehydration solution to constant weight after rehydration experiments. The end of the drying experiments is estimated according to the assumed 85% moisture content dried to 13%, resulting in inaccurate moisture contents after drying. After 300 min of rehydration, the shiitake mushrooms’ dry basis moisture contents rise from 0.13–0.31 to 2.42–5.54. Among all the drying processes, mushrooms dried at 338.15 K have the lowest dry basis moisture content. The rehydration ratios under various drying processes follow a similar trend to the dry base moisture contents. The rehydration ratios under different drying processes increase from 1 to 3.03–5.32 after rehydration. The rehydration ratio of the shiitake mushroom dried at 338.15 K is the smallest, and the rehydration ratio of the shiitake mushroom dried at 318.15 K is among that of shiitake mushrooms dried under different VTCD processes. The rehydration ratios of VTCD exceed 2 within 30 min of rehydration, and similar results have been obtained in the literature [29]. The rehydration speed of shiitake mushrooms after VTCD is fast during the initial rehydration stage, possibly due to the porous structure and low shrinkage rate of the mushrooms. Similar findings have also been reported in the literature [64].

The initial moisture contents of mushroom samples range from 4.58 to 7.61 due to the individual variances in shiitake mushrooms. Combined with Figure 11a and Table 5, the dry basis moisture contents after rehydration are all lower than the initial moisture contents, indicating irreversible cell damage and dislocation during the drying process. The significant shrinkage of the capillary and collapse of the dense tissue structure reduce hydrophilicity, so it cannot wholly absorb enough water [26]. Since the mushroom samples’ initial properties cannot be entirely consistent, it is difficult to eliminate the interference of different initial properties in the rehydration experimental results. Therefore, a numerical model combining drying and rehydration is established, which unifies the material properties and is used to analyze the effect of VTCD processes on mushroom rehydration performance.

Because the dry matter content determination is a destructive analysis [65], the time-dependent solid leakage rate during rehydration is unknown, and only the DHC is measured after rehydration. According to Table 5, the DHCs of rehydrated shiitake mushrooms under different drying processes are close and high (the average DHC is 0.77). Therefore, solid leakage is eventually not considered.

#### 3.2.2. Rehydration Model Related to Variable-Temperature Convective Drying Conditions

The Peleg equation (Equation (29)) is applied to the experimental data of the rehydration curve. Table 6 shows the *K*_1_ and *K*_2_ values for dried shiitake mushrooms under different drying conditions. The adjusted R-square values vary from 0.9906 to 0.9994, while reduced Chi-square values range from 0.0012 to 0.0125. These values indicate an excellent concordance with the experimental data, implying that Peleg’s model could reasonably describe the rehydration kinetics of shiitake mushrooms under examined conditions.

The Peleg rate constant *K*_1_ relates to the initial water absorption rate, and its reciprocal can be likened to a diffusion coefficient [66]. The lower the *K*_1_, the higher the water absorption rate at the beginning of rehydration. Table 6 shows that *K*_1_ ranges from 1.05 to 17.71 min under the 19 drying processes. Shiitake mushrooms dried at 338.15 K have the highest *K*_1_, indicating the lowest water absorption rate during rehydration. The Peleg capacity constant *K*_2_ refers to the maximum attainable moisture content during rehydration, i.e., equilibrium moisture content [67]. Under experimental drying conditions, *K*_2_ is between 0.18 and 0.39. Although the 338.15 K CTCD process has the shortest total drying time, the moisture content is the lowest when rehydration reaches equilibrium. This is because the high temperature throughout the drying process causes intensive structural changes and reduces the extent of rehydration.

Table 7 shows the ANOVA result for the reduced quadratic model of Peleg rate constant *K*_1_. The model F-value of 16.25 implies the model is significant. There is a 0.01% chance that an F-value this large is caused by noise. *p*-values less than 0.05 indicate that model terms are significant. B, BC, A^2^, B^2^, C^2^ are significant terms. B, i.e., drying time at 318.15 K, is the most critical factor affecting the Peleg rate constant *K*_1_ of shiitake mushrooms during rehydration. The model’s significance and adequacy are collaborated by the coefficient of determination (R^2^) of 0.91. As reported in Table 8, an F value of 7.91 implies that the model for *K*_2_ is significant. The chance that the F value is caused by noise is 0.42%. B, A^2^, and A^2^B are significant model terms. B, i.e., the drying time at 318.15 K, is the most critical factor affecting *K*_2_. R^2^ (0.89) collaborates on the significance and adequacy of the model.

The equation in terms of coded factors is used to predict the response for given levels of each factor. High levels (+1) and low levels (−1) of the factors (A, B, and C) are assigned by default. The coded equation helps identify the relative impacts of factors based on their coefficients. The equations for *K*_1_ and *K*_2_ in terms of coded factors are given by Equations (33) and (34). They establish the connection between VTCD processes and rehydration kinetics.

(33) $$K1=5.82586-0.51325\times A+3.02139\times B-1.4353\times BC-2.91671\times A^{2}+2.03687\times B^{2}+2.22422\times C^{2}$$

(34) $$\begin{array}{l} K2=0.236811+0.0088125\times A+0.047875\times B+0.0071875\times C\\ +0.0134\times AC-0.017175\times BC-0.0340987\times A^{2}+0.0176513\times C^{2}-0.03805\times A^{2}B \end{array}$$

#### 3.2.3. Validation of the Numerical Rehydration Model

**Figure 12:**
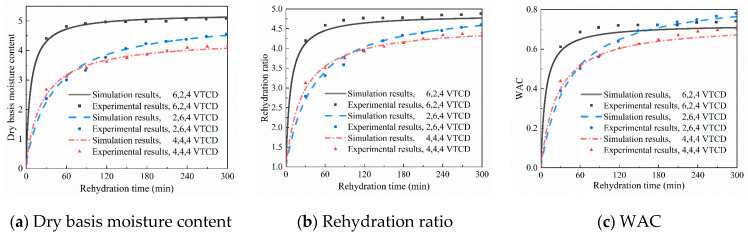
Comparison between simulated and experimental results for rehydration.

Representative experiments are selected to verify the rehydration model’s accuracy. Since the initial conditions significantly affect the experimental results, the model’s input parameters (including ambient conditions, drying conditions, and the mushroom moisture content before and after drying) are expressed using the values obtained from corresponding experiments. Figure 12 compares the simulated and experimental rehydration performance, including dry basis moisture content, rehydration ratio, and WAC. The results show that the model results agree with the actual situation. Therefore, the initial conditions, except drying processes, are unified in the model, and the comprehensive influence of VTCD processes on rehydration performance and drying time is analyzed.

#### 3.2.4. Assessment of Rehydration after Variable-Temperature Convective Drying

**Figure 13:**
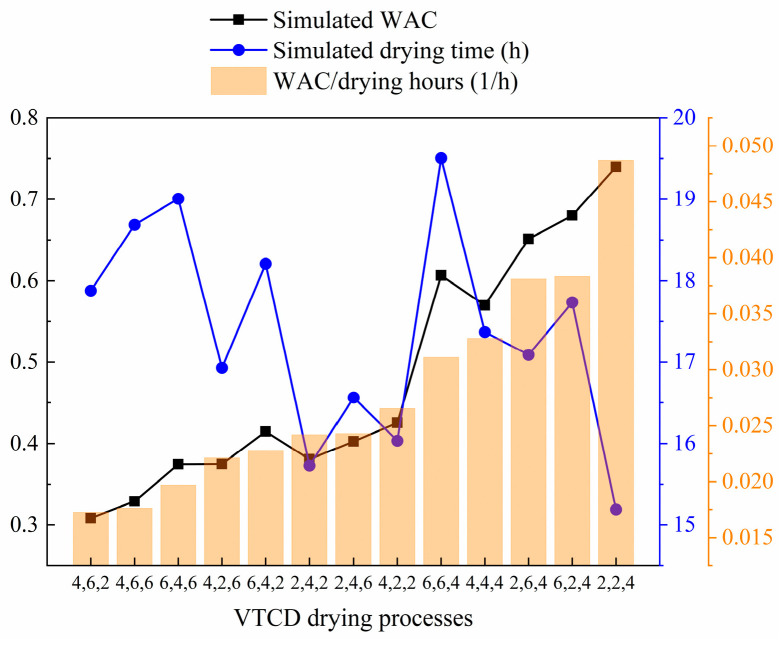
The effect of VTCD processes on WAC and total drying time.

Shiitake mushroom enzymatic and non-enzymatic reactions are primarily influenced by drying temperature and drying time [11], and WAC is related to chemical composition [62]. Therefore, shiitake mushroom WACs are simulated using different VTCD processes. Figure 13 illustrates how VTCD processes affect WAC and total drying time. The simulation input parameters are the same as those in Table 2. The results show that, among the 13 VTCD processes, the highest WAC is 0.74 (2,2,4 VTCD), and the lowest WAC is 0.31 (4,6,2 VTCD), while the longest total drying time is 19.51 h (6,6,4 VTCD), and the shortest is 15.19 h (2,2,4 VTCD). Generally speaking, the greater the WAC, the less damage the mushroom tissue sustains during drying and the better the drying quality. In large-scale factory production, the drying efficiency improves as the total drying time decreases. Therefore, we hope to obtain a drying process with high mushroom WAC and short total drying time to achieve a win–win situation between drying quality and drying efficiency. Figure 13 shows the VTCD processes sorted according to the ratio of WAC to total drying time, which can be referred to as the actual production of shiitake mushroom drying.

## 4. Conclusions

Variable-temperature convective drying has less variation in shiitake mushroom temperature at the initial stage than with constant-temperature convective drying. Moreover, variable-temperature convective drying has a moderate shrinkage rate and less variation in evaporation rate. It can simultaneously achieve a high evaporation rate and a low evaporation rate gradient.

As for the drying load characteristics, variable-temperature convective drying demonstrates moderate total drying time (16.89 h) and better enthalpy stability, reducing the maximum drying load by 58.84% compared to 338.15 K constant-temperature convective drying.

The rehydration kinetics of shiitake mushrooms are modelled as a function of the drying time of each temperature zone in variable-temperature convective drying. All variable-temperature dried mushrooms exhibit superior rehydration performance better than those dried at a constant temperature of 338.15 K. The drying time at 318.15 K has the most significant impact on the rehydration kinetic parameters. The variable-temperature convective drying process with moderate drying time (4 h) at 328.15 K and short drying time (2 h) at low temperature (318.15 K) displays the best performance weighing the rehydration capacity and drying efficiency.

In conclusion, variable-temperature convective drying outperforms constant-temperature convective drying regarding dehydration characteristics, energy supply–demand matching, and rehydration performance. Nevertheless, further research is needed to explore other characteristic changes of shiitake mushrooms during variable-temperature convective drying processes, such as color, flavor compounds, and nutrients.

## Figures and Tables

**Table 1:** Factors and levels in BBD.

Factor (h)	−1 Level	0 Level	1 Level
A: Drying time at 308.15 K	2	4	6
B: Drying time at 318.15 K	2	4	6
C: Drying time at 328.15 K	2	4	6

**Table 2:** Parameters of the drying model.

Parameter	Value	Unit	Description
*cp*_s_	1500 [51]	J/(kg·K)	Solid matrix heat capacity
*E*_m_	4 × 10^5^ [50]	Pa	Mushroom elastic modulus
*H*	2.37055 × 10^6^	J/kg	Evaporation latent heat
*K*	1	1/s	Non-equilibrium evaporation rate constant
*k*_s_	0.1 [51]	W/(m·K)	Solid matrix thermal conductivity
*p*_0_	101,325	Pa	Ambient pressure
*RH*_0_	0.8	-	Ambient relative humidity
*S*_il_	0.05 [38]	-	Irreducible liquid phase saturation
*S*_l,0_	0.62	-	Initial water saturation
*T*_0_	293.15	K	Ambient temperature
*u*_in_	3.0	m/s	Freestream velocity
*por*	0.95 [50]	-	Initial porosity
*α*	2.5 × 10^−6^ [50]	1/K	Thermal expansion coefficient
*κ*	1 × 10^−14^	m^2^	Absolute permeability of shiitake mushroom
ν	0.49	-	Poisson’s ratio
*ρ*_s_	1591 [50]	kg/m^3^	Solid matrix density

**Table 3:** Initial conditions.

Physical Quantity	Porous Medium Domain	Air Free Flow Domain	Description
*U*	0	0	Velocity
*P*	0	0	Pressure
*T*	*T*_0_	*T*_0_	Temperature
*c*_l,0_	*S*_l0_ × *por* × *ρ*_l_/*Mn*_l_	0	Liquid water concentration
*c*_v,0_	*c*_v,sat,0_ × (1 − *S*_l,0_) × *por*	*c*_v,sat,0_ × *RH*_0_	Water vapor concentration
*U*	0	-	Displacement
$\frac{\partial U}{\partial t}$	0	-	Structural velocity

**Table 4:** Grid convergence test.

Grid	Number of Cells	Wet-Basis Moisture Content	Relative Error	GCI
1	12,645	0.46219		
2	20,633	0.46147	0.0016	0.0030
3	50,163	0.45735	0.0090	0.0075

**Table 5:** Experimental results of initial dry basis moisture content and DHC after rehydration.

Drying Processes	Initial Dry Basis Moisture Content	DHC
6,4,6 VTCD	4.58 ± 0.52	0.76 ± 0.02
4,6,2 VTCD	4.75 ± 0.81	0.69 ± 0.02
4,6,6 VTCD	4.75 ± 0.47	0.71 ± 0.04
6,6,4 VTCD	5.10 ± 0.59	0.77 ± 0.002
338.15 K CTCD	5.31 ± 0.84	0.75 ± 0.05
2,6,4 VTCD	5.77 ± 0.44	0.79 ± 0.06
4,2,6 VTCD	5.78 ± 1.33	0.81 ± 0.06
4,4,4 VTCD	6.04 ± 1.15	0.79 ± 0.01
2,2,4 VTCD	6.31 ± 1.51	0.75 ± 0.06
2,4,2 VTCD	6.73 ± 1.63	0.73 ± 0.03
318.15 K CTCD	6.86 ± 1.18	0.77 ± 0.06
6,4,2 VTCD	6.92 ± 1.14	0.80 ± 0.02
4,2,2 VTCD	7.38 ± 1.38	0.79 ± 0.05
2,4,6 VTCD	7.54 ± 0.32	0.75 ± 0.01
6,2,4 VTCD	7.61 ± 0.62	0.78 ± 0.02

**Table 6:** Peleg constants for the rehydration of dried shiitake mushrooms under different drying conditions.

RUN	Drying Process	A Drying Time at 308.15 K (h)	B Drying Time at 318.15 K (h)	C Drying Time at 328.15 K (h)	*K*_1_ (min)	Standard Error	*K*_2_	Standard Error	Reduced Chi-Sqr	Adj. R-Square
1	VTCD	4	6	2	15.29	0.55	0.31	0.0037	0.0013	0.9981
2		4	4	4	6.98	0.67	0.27	0.0054	0.0097	0.9906
3		2	4	2	5.16	0.18	0.22	0.0015	0.0018	0.9988
4		4	2	6	7.76	0.42	0.23	0.0032	0.0047	0.9965
5		2	4	6	4.17	0.19	0.21	0.0017	0.0030	0.9983
6		6	4	2	5.48	0.24	0.21	0.0019	0.0034	0.9980
7		4	4	4	6.79	0.47	0.25	0.0037	0.0059	0.9950
8		6	2	4	1.05	0.06	0.21	0.0007	0.0012	0.9994
9		6	4	6	5.73	0.32	0.25	0.0027	0.0036	0.9971
10		4	4	4	4.43	0.38	0.24	0.0034	0.0076	0.9943
11		2	2	4	3.86	0.14	0.18	0.0012	0.0028	0.9988
12		4	2	2	5.31	0.32	0.18	0.0025	0.0092	0.9959
13		4	4	4	4.92	0.33	0.22	0.0028	0.0062	0.9959
14		4	6	6	12.00	0.93	0.29	0.0066	0.0064	0.9920
15		2	6	4	9.03	0.53	0.20	0.0037	0.0081	0.9952
16		6	6	4	5.84	0.39	0.22	0.0031	0.0068	0.9955
17		4	4	4	6.00	0.50	0.21	0.0039	0.0125	0.9926
18	CTCD	318.15 K			7.37	0.37	0.20	0.0027	0.0058	0.9968
19		338.15 K			17.71	1.15	0.39	0.0079	0.0027	0.9940

**Table 7:** ANOVA for the reduced quadratic model of the Peleg rate constant *K*_1_.

Source	Sum of Squares	df	Mean Square	F-Value	*p*-Value	
Model	154.22	6	25.70	16.25	0.0001	significant
A—Drying time at 308.15 K	2.11	1	2.11	1.33	0.2753	
B—Drying time at 318.15 K	73.03	1	73.03	46.16	0.0001	
BC	8.24	1	8.24	5.21	0.0456	
A^2^	35.82	1	35.82	22.64	0.0008	
B^2^	17.47	1	17.47	11.04	0.0077	
C^2^	20.83	1	20.83	13.17	0.0046	
Residual	15.82	10	1.58			
Lack of Fit	10.77	6	1.79	1.42	0.3826	not significant
Pure Error	5.05	4	1.26			
Cor Total	170.04	16				

**Table 8:** ANOVA for the reduced cubic model of the Peleg capacity constant *K*_2_.

Source	Sum of Squares	df	Mean Square	F-Value	*p*-Value	
Model	0.0184	8	0.0023	7.91	0.0042	significant
A—Drying time at 308.15 K	0.0006	1	0.0006	2.13	0.1824	
B—Drying time at 318.15 K	0.0092	1	0.0092	31.46	0.0005	
C—Drying time at 328.15 K	0.0004	1	0.0004	1.42	0.2679	
AC	0.0007	1	0.0007	2.46	0.1551	
BC	0.0012	1	0.0012	4.05	0.0790	
A^2^	0.0049	1	0.0049	16.84	0.0034	
C^2^	0.0013	1	0.0013	4.51	0.0664	
A^2^B	0.0029	1	0.0029	9.94	0.0136	
Residual	0.0023	8	0.0003			
Lack of Fit	0.0001	4	0.0000	0.06	0.9904	not significant
Pure Error	0.0022	4	0.0005			
Cor Total	0.0208	16				

**Figure A1:**
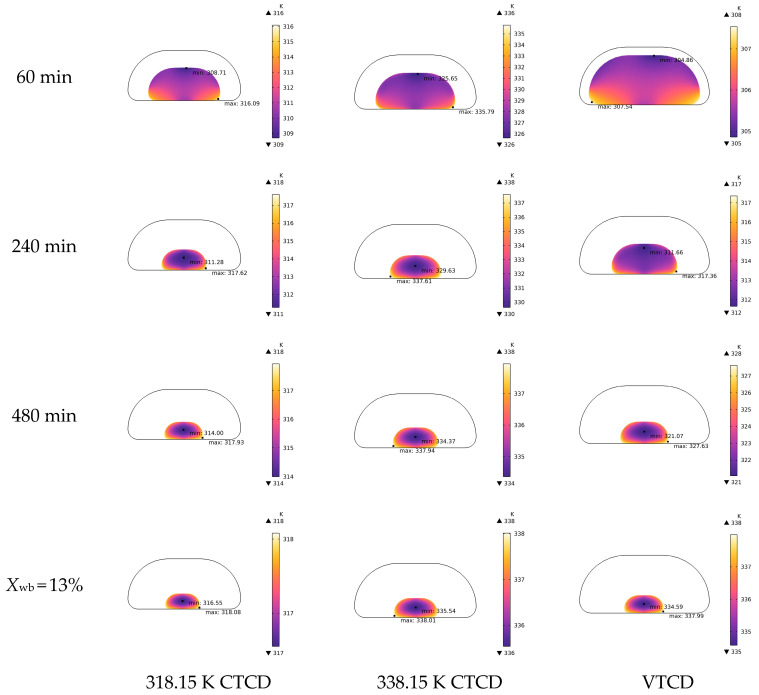
Temperature distribution of shiitake mushrooms in VTCD and CTCD processes.

**Figure A2:**
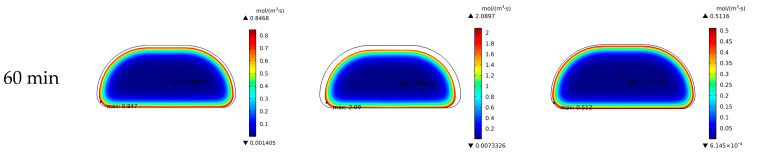

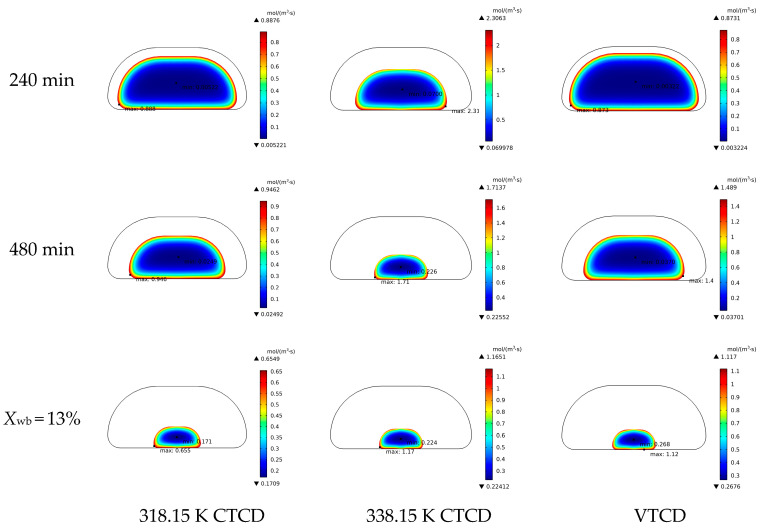
Evaporation rate distribution of shiitake mushrooms in VTCD and CTCD processes.

## Data Availability

The original contributions presented in the study are included in the article, further inquiries can be directed to the corresponding authors.

## Supplementary Materials

The following supporting information can be downloaded at: https://www.mdpi.com/article/10.3390/foods13213356/s1.

## Appendix A. Formula Derivation of Mathematical Model Combining Drying and Rehydration

The mushroom’s density is obtained by the density and volume fraction of each phase:

(A1)
$$\rho_{m,0}=\rho_{s}x_{s,0}+\rho_{l,0}x_{l,0}+\rho_{\mathrm{ma},0}\left(1-x_{s,0}-x_{l,0}\right)$$

(A2) $$\rho_{m,d}=\frac{\rho_{s}x_{s,0}}{1-SR}+\frac{\rho_{l}x_{l,0}MR}{1-SR}+\rho_{g}\left(1-\frac{x_{s,0}}{1-SR}-\frac{x_{l,0}MR}{1-SR}\right)$$

where *ρ*_m,0_ and *ρ*_m,d_ represent the mushroom density before and after drying, respectively, kg/m^3^; *ρ*_s_, *ρ*_l,0_, and *ρ*_ma,0_ are the density of solid, liquid, and gas phases in shiitake mushroom before drying, respectively, kg/m^3^; *ρ*_l_ and *ρ*_g_ are the density of liquid phase and gas phase in shiitake mushroom after drying, kg/m^3^; *x*_s,0_ and *x*_l,0_ are the initial volume fraction of solid matrix and liquid water; *MR* is the moisture ratio; *SR* is the volume shrinkage ratio.

The mushroom mass ratio in the drying process is calculated by density ratio and volume shrinkage ratio.

(A3)
$$\frac{m_{0}}{m_{d}}=\frac{\rho_{m,0}V_{m,0}}{\rho_{m,d}V_{m,d}}=\frac{\rho_{m,0}}{\rho_{m,d}}\times \frac{1}{1-SR}$$

The dry basis moisture contents are calculated from rehydration experimental results, and the rehydration kinetic parameters are fitted. Rehydration indices are derived.

(A4)
$$X_{\mathrm{db},r}=\frac{m_{r}-m_{s,r}}{m_{s,r}}=\frac{m_{r}-DHC\times m_{s,d}}{DHC\times m_{s,d}}=\frac{m_{r}}{DHC\times m_{s,d}}-1$$

(A5)
$$X_{\mathrm{db},r}=X_{\mathrm{db},d}+\frac{t}{K_{1}+K_{2}\times t}$$

(A6)
$$X_{\mathrm{wb}}=\frac{X_{\mathrm{db}}}{1+X_{\mathrm{db}}}$$

(A7)
$$RR=\frac{m_{r}}{m_{d}}=\frac{m_{l,r}+m_{s,r}}{m_{l,d}+m_{s,d}}=\frac{m_{l,r}+DHC\times m_{s,d}}{m_{l,d}+m_{s,d}}=\frac{X_{\mathrm{db},r}+1}{X_{\mathrm{db},d}+1}DHC$$

(A8) $$WAC=\frac{m_{r}\left(1-s_{r}\right)-m_{d}\left(1-s_{d}\right)}{m_{0}\left(1-s_{0}\right)-m_{d}\left(1-s_{d}\right)}=\frac{m_{r}X_{\mathrm{wb},r}-m_{d}X_{\mathrm{wb},d}}{m_{0}X_{\mathrm{wb},0}-m_{d}X_{\mathrm{wb},d}}=\frac{RRX_{\mathrm{wb},r}-X_{\mathrm{wb},d}}{\frac{m_{0}}{m_{d}}X_{\mathrm{wb},0}-X_{\mathrm{wb},d}}$$

where *m*_0_, *m*_d_, and *m*_r_ are the shiitake mushroom mass before drying, after drying, and after rehydration, respectively, kg; *V*_m,0_ and *V*_m,d_ represent the shiitake mushroom volumes before and after drying, m^3^; *DHC* is the dry matter holding capacity during rehydration; *RR* is the rehydration ratio; *m*_l,r_ and *m*_s,r_ are the mass of liquid water and solid matrix in mushroom after rehydration, kg; *m*_l,d_ and *m*_s,d_ are the mass of liquid water and solid matrix in mushroom after drying, kg; *X*_db,r_ and *X*_db,d_ are the dry basis moisture content after and before rehydration; *WAC* is the water absorption capacity during rehydration; *s*_0_, *s*_d_, and *s*_r_ are the dry matter content of initial, dried, and rehydrated mushrooms, respectively; *X*_wb,0_, *X*_wb,d_, and *X*_wb,r_ represent the wet-basis moisture content of initial, dried, and rehydrated mushrooms, respectively.
