# Supplementary material for: Numerical Study on the Variable-Temperature Drying and Rehydration of Shiitake
Source: Foods. 2024 Oct 23;13(21):3356. doi: 10.3390/foods13213356 (PMC11545107; doi:10.3390/foods13213356)
Supplement: Supplementary file 1 [file foods-13-03356-s001.zip › foods-3259511-supplementary.pdf]

SUPPLEMENTARY MATERIAL

Title: Numerical Study on the Variable-Temperature Drying and Rehydration of Shiitake

Authors: Lizhe Zhang , Long Jiang , Meriem Adnouni , Sheng Li , Xuejun Zhang*

1. The relationship between generalised Maxwell parameters and drying conditions

$$\sigma_{\mathrm{q}}(t)=\sum_{1}^{3}\varepsilon_0 G_{\mathrm{vm,i}}\exp\left(-\frac{t}{\tau_{\mathrm{i}}}\right)+\sigma_{\mathrm{r}} \tag{S1}$$

$$\begin{aligned}
\sigma_{\mathrm{r}} &= 1000\times\exp\left[7.0824 - 3.1220u + 0.0013(T\text{-}273.15) - 0.5244X_{\mathrm{db}}\right]\\
G_{\mathrm{vm,1}} &= 1000\times\exp\left[5.7736 - 1.9791u - 0.0145(T\text{-}273.15) - 0.5825X_{\mathrm{db}}\right]\\
G_{\mathrm{vm,2}} &= 1000\times\exp\left[5.6309 - 1.8681u - 0.0136(T\text{-}273.15) - 0.4460X_{\mathrm{db}}\right]\\
G_{\mathrm{vm,3}} &= 1000\times\exp\left[4.1252 - 1.1153u - 0.0069(T\text{-}273.15) - 0.2813X_{\mathrm{db}}\right]\\
\tau_1 &= \exp\left[-1.9287 + 0.7270u + 0.0025(T\text{-}273.15) + 0.1585X_{\mathrm{db}}\right]\\
\tau_2 &= \exp\left[-0.1302 + 1.1595u + 0.0046(T\text{-}273.15) + 0.1699X_{\mathrm{db}}\right]\\
\tau_3 &= \exp\left[1.5573 + 1.7933u + 0.0099(T\text{-}273.15) + 0.1287X_{\mathrm{db}}\right]
\end{aligned} \tag{S2}$$

where $\sigma_q(t)$ is the viscoelastic stress at time t, Pa; ε_0 is the initial strain with a value of 0.7; $G_{vm,i}$ is the shear modulus for the i-th Maxwell branch, Pa; τ_i is the relaxation time for the i-th Maxwell branch, s; σ_r is the residual stress, Pa; u is the air velocity, m/s; T is the temperature, K; X_{db} is the dry basis moisture content.

2. The calculation and meanings of the statistical parameters

Coefficient of Determination (R^2), which assesses the goodness-of-fit of the model. It is given by:

$$R^2 = 1-\frac{\sum\left(Y_{\mathrm{exp}}-Y_{\mathrm{pred}}\right)^2}{\sum\left(Y_{\mathrm{exp}}-\overline{Y}_{\mathrm{exp}}\right)^2} \tag{S3}$$

where Y_{exp} represents the experimental values, Y_{pred} are the predicted values, and $\overline{Y}_{\mathrm{exp}}$ is the mean of the experimental values.

Root Mean Square Error (RMSE), which measures the differences between predicted and observed values:

$$RMSE = \sqrt{\frac{\sum\left(Y_{\mathrm{exp}}-Y_{\mathrm{pred}}\right)^2}{n}} \tag{S4}$$

where n is the number of observations.

Mean Absolute Error (MAE), which provides the average of absolute errors between the predicted and experimental values:

$$MAE = \frac{\sum\left|Y_{\mathrm{exp}}-Y_{\mathrm{pred}}\right|}{n} \tag{S5}$$

Reduced Chi-Square (χ^2), which tests the goodness-of-fit of the model:

$$\chi^2 = \frac{\sum\frac{\left(Y_{\mathrm{exp}}-Y_{\mathrm{pred}}\right)^2}{Y_{\mathrm{pred}}}}{n-p} \tag{S6}$$

where p is the number of parameters in the model.

Adjusted R-Square (Adj. R^2), which adjusts the R^2 value based on the number of predictors in the model:

$$Adj.R^2 = 1 - \left[\frac{(1 - R^2)(n - 1)}{n - p - 1} \right] \tag{S7}$$

Standard Error (SE), which represents the standard deviation of the error term.

$$SE = \sqrt{\frac{\sum (Y_{\text{exp}} - Y_{\text{pred}})^2}{n(n-1)}} \tag{S8}$$
